# Trusted contraception information sources for individuals with opioid use disorder

**DOI:** 10.1111/1475-6773.14075

**Published:** 2022-10-10

**Authors:** Lauren Sobel, Yeon Woo Lee, Katharine White, Elisabeth Woodhams, Elizabeth Patton

**Affiliations:** ^1^ Department of Obstetrics & Gynecology Brigham & Women's Hospital/Harvard Medical School Boston Massachusetts USA; ^2^ Department of Obstetrics & Gynecology Boston Medical Center/Boston University Boston Massachusetts USA

**Keywords:** chronic disease, contraception, opioid use disorder, substance use

## Abstract

**Objective (Study Question):**

To identify trusted sources of contraception information among pregnancy‐capable individuals with opioid use disorder (OUD).

**Data Sources/Study Setting:**

We conducted interviews between October 2018 and January 2019 at Boston Medical Center, a university‐based tertiary care center.

**Study Design:**

Data were drawn from semi‐structured qualitative interviews with a convenience sample of 20 pregnant or recently pregnant individuals with OUD. We used the Ottawa Decision Support Framework, a health decision making conceptual model, to structure our interviews. We analyzed the data using inductive and deductive coding.

**Data Collection/ Extraction Methods:**

Not applicable.

**Principal Findings:**

Pregnancy‐capable individuals who use opioids value friends who are not actively using opioids, including peers in recovery homes, as trusted sources of contraception information. They also value internet resources, including websites recommended by clinicians and social media posts, and established clinical providers as reliable sources of contraception information in ways that emulate individuals with other chronic medical conditions.

**Conclusion:**

These sources of contraception information may explain some trends in contraceptive use among individuals with OUD, inform nonstigmatizing contraceptive counseling, and serve as a foundation for improved decision support.

**What is known on this topic**

- People with chronic conditions are at increased risk of having unintended pregnancies
- Existing research suggests that persons with opioid use disorder have unique patterns of contraception use
- Knowledge on contraception information sources valued by persons with opioid use disorder is limited

**What this study adds**

- Persons with opioid use disorder value peers who were not actively using opioids as trusted sources of contraception information
- Persons with opioid use disorder value contraceptive counseling in which clinicians center their lived contraception experiences and personal goals

## 1 INTRODUCTION

People with chronic conditions are at increased risk of having unintended pregnancies. Yet these individuals often require multidisciplinary and proactive pregnancy planning for optimal maternal and fetal health.^1^, ^2^ While 45% of pregnancies in the general population are considered unwanted, or unintended, one study found that participants with opioid use disorder (OUD) were nearly twice as likely to report this outcome.^3^, ^4^

Contraceptive use patterns differ between people with chronic conditions, including diabetes, cardiovascular disease, asthma, HIV, and OUD and those without.^1^, ^5^, ^6^ Similar to persons with HIV, individuals with OUD make their contraceptive decisions in the context of a highly stigmatized chronic condition, societal views that they should not become pregnant, and a history of coercive contraceptive practices.^5^, ^7^, ^8^ Given this systems‐level devaluation of their reproductive autonomy compounded by the high‐risk nature of pregnancy for many people with chronic conditions, individuals with OUD may not feel comfortable explicitly stating their pregnancy intentions to a clinician.^9^ Contraceptive decision making and use for persons with OUD also occurs in the context of perceived and actual decreased fertility, sexual partner power dynamics, and the intersection of common contraceptive side effects and active opioid use.^10^, ^11^, ^12^ Standard assessments of contraceptive use among women with substance use disorder (SUD) are limited. Yet one systematic review reported that 56% of women with SUD utilized contraception, compared to 81% individuals without SUD.^13^

Patients make contraceptive decisions informed by the recommendation of medical professionals, but also with the input of partners, friends, family, and social media networks.^14^, ^15^, ^16^, ^17^, ^18^ Yet little is known about the contraceptive information sources valued by individuals with OUD. As such, we sought to understand patient perspectives on trusted sources of contraception information for pregnancy‐capable individuals with OUD.

## 2 METHODS

As part of our broader exploration of contraceptive decision making among pregnancy capable individuals with OUD, we conducted semi‐structured qualitative interviews between October 2018 and January 2019 at Boston Medical Center, a university‐based care center.

We recruited our sample from Project RESPECT (Recovery, Empowerment, Social Services, Prenatal care, Education, Community and Treatment), a specialized outpatient obstetric clinic for patients with substance use disorder (SUD), the SOFAR (Supporting Our Families through Addiction and Recovery) program, an outpatient clinic for parents in SUD recovery and their children, and an inpatient maternal fetal medicine service, where patients are admitted for inpatient medication for OUD titration. Participants were recruited via provider referral, self‐referral in response to flyers, and referral by prior participants. Eligibility criteria were (1) currently pregnant or pregnant within one year regardless of outcome, (2) self‐identified OUD, (3) 18 years old or older, and able to consent, and (4) English speaking. We continued recruitment until we reached thematic saturation. We provided a $50 gift card for participation.

We obtained written informed consent prior to each interview. Interviewers (LS and YWL) both identified as female and were an OB‐GYN resident and OB‐GYN medical student with prior qualitative interviewing experience. They were not directly involved in the participants' medical care. They conducted in person interviews in the clinical environment. Interviews were audio recorded and professionally transcribed. Interviewers took field notes after the conclusion of each interview.

We structured our interview guide around the Ottawa Decision Support Framework, an internationally recognized conceptual model describing health decision making.^19^ Interviews began with the open‐ended statement: “Tell me about the contraceptive methods you've used in the past” followed by questions related to the Ottawa Decision Support Framework decisional needs. We specifically explored trusted information sources in our interview guide. This topic was initiated with the anchor question: “Tell me about where you get your birth control information”. This question was followed by probing questions, including: “Do you feel that one of these sources has been most helpful to you?” and “Which one of these do you think is the best source of information?”. We administered a demographic survey after the interview which we entered into REDCap.^20^

LS and EWP independently analyzed five transcripts using inductive and deductive coding to develop a codebook. Each subsequent transcript was coded by LS and either EWP or YWL. They met regularly to ensure coding consistency, resolve discrepancies through consensus, and iteratively revise the codebook. We used NVivo 11 software to manage our data and facilitate coding. The Boston University Institutional Review Board approved this study.

## 3 RESULTS

We screened 131 individuals and approached 58 for study participation. Twenty‐two individuals ultimately completed interviews. Patients most often declined participation due to time constraints or withdrawal symptoms. Two interviews were not included in the final analysis because the identified their primary substance use to be cocaine or alcohol rather than opioids. (Table 1). Four primary contraception information sources valued by individuals with OUD emerged.

**TABLE 1:** Characteristics of interview participants (*N* = 20)

Characteristic	*n* (%) or mean ± standard deviation (range)
Age (years)	31.7 ± 4.91 (21–40)
Ethnicity and race	
Non‐Hispanic White	13 (65%)
Non‐Hispanic Black	3 (15%)
Hispanic	4 (20%)
Relationship status	
Single	5 (25%)
In a relationship	11 (55%)
Married	1 (5%)
Other	3 (15%)
Highest level of education	
Middle school	1 (5%)
Highschool or equivalent	6 (30%)
Some college	11 (55%)
College	2 (10%)
Age of first non‐Rx Opioid use (years)	19.29 ± 4.43 (13–29)
Last non‐Rx Opioid use (months)	22.68 ± 25.87 (0–96)
Medication assisted treatment	
Methadone	11 (55%
Buprenorphine and naloxone	6 (30%)
Buprenorphine	0 (0%)
None	3 (15%)
Longest period on MAT (months)	19.68 ± 20.03 (0–84)
Lifetime overdoses	3.72 ± 4.87 (0–15)
Pregnancies	4.25 ± 2.81 (1–12)
Living children^a^	2.05 ± 1.43 (0–6)
Number of children in physical custody	0.94 ± 1.11 (0–4)
Current case open with DCF	
Yes	11 (55%)
No	9 (45%)

Abbreviations: DCF, department of children and families; MAT, medication assisted treatment; Rx, prescription.

^a^ *N* = 19 who had at least one birth.

### 3.1 Trusted information source 1: Friends who have never used opioids and those stable in recovery

Participants commonly cited friends as sources of contraception information. They noted that friends who had never used opioids, as well as those who were stable in their recovery, modeled admirable preventative health care behaviors. This resulted in more value being placed on the health information they provided: *“My girlfriends that like don't think it's okay to shoot heroin with your kid in the next room… You know? I consider them my normal friends. They're all on birth control… [and take it] at a certain time every day… and it's like clockwork. I listen to them”* (Participant 7). They also described that friends who regularly engaged with social media were less likely to be actively using opioids: *“A lot of my Facebook friends will post about their experiences with [contraception]… not everyone is on Facebook. Not friends [who use opioids]—they're not gonna post on Facebook.”* (Participant 19) Given these attitudes, participants viewed contraceptive experiences shared on social media as a reliable source of contraception information.

### 3.2 Trusted information source 2: Recovery home peers

Multiple participants cited recovery home peers as trusted sources of contraception information. Recovery homes are group living arrangements that provide a structured environment for individuals in the immediate postacute phase of substance use as a transition into independent living.^21^ Multiple participants described formal sexually transmitted infection education, yet only two participants cited formal contraception education in their recovery programs: *“I remember at the program I was at before this one – a nurse came in and talked to us about all the different [contraception] options.”* (Participant 16) More commonly, participants described peers in their recovery home sharing both negative and positive contraception experiences. They also noted that some recovery homes had contraception trends based on the shared experiences: *“I mean, everyone in the house had Nexplanon. So I knew that was kind of like a trending thing… A girl came home with a bandage on her arm and I was like, what's that from? And she's like oh, it's uh, the implant. I was like, what's the implant? And she told me about it and then I was like wow, that sounds great.”* (Participant 21)

### 3.3 Trusted information source 3: Medical professionals

Participants identified their obstetrician/ gynecologists, addiction medicine specialists, and their child's pediatrician with whom they often had long‐standing relationships as reliable sources of contraception information, regardless of their training in contraception counseling: *“And also [my daughter's] pediatrician will ask me [about birth control] because she knows us*… *I don't mind talking to her about it at all.”* (Participant 16). Yet, even among clinicians they identified as trusted sources of contraception information, they questioned their ability to counsel on contraceptive side‐effects and interactions in the context of their chronic condition. *“Doctors… just kind of know how it works… but like they don't really get the side effects that the patients like us get.”* (Participant 19). The side effects, most commonly referred to, included weight gain with contraception use, which for some can trigger a relapse, personal hygiene related to bleeding patterns in the setting of housing instability, and interactions with mood stabilizing medications.^9^

### 3.4 Trusted information source 4: Personal experience and research

Participants strongly desired that their clinicians understood the importance of both their personal contraceptive experiences and their own research. Participants drew from their personal experience with contraception in the context of their chronic disease: “*I've tried pretty much all of the birth control that there is out there. I can tell you what my body has done for each birth control, so, that's kind of where I've gotten most of my information.”* (Participant 18). They valued their ability to identify evidence‐based information sources and recognize misinformation. They also appreciated when clinicians recommended reliable contraception information sources: *“I don't look at posts where people aren't of some kind of [medical] background*… *I like to go on the websites my doctors tell me”* (Participant 4). When participants felt clinicians did not value their research and experience, they described less trust in the medical information provided: *“If we were to talk about breastfeeding with my [Hepatitis] C, I would shut a doctor down if they told me it's not a good idea and not trust them on anything else because I've done a ton of online research about it.”* (Participant 2).

## 4 DISCUSSION

Understanding the contraception information sources valued by persons with OUD provides a foundation for improved patient‐centered and nonstigmatizing counseling, as well as multiple points for evidence‐based interventions.

Clinicians use a variety of communication strategies when engaging in contraception counseling. Directive counseling can lead to experiences with clinician‐based reproductive coercion to use highly effective contraceptive methods, which contributes to health care system distrust.^22^ Data supports grounding contraception counseling for individuals with OUD in an open and supportive narrative about reproductive goals, which align with their pursuit of sustained recovery.^9^, ^22^, ^23^ Structured counseling models such as the PATH framework and PHI CARE may support the elicitation of patients' family planning values.^24^, ^25^ Consistent with this approach, participants describe a preferred contraception counseling dynamic, which centers on their lived contraceptive experiences, as well as their contraceptive values and goals.

People with chronic conditions often seek condition‐specific contraceptive counseling. Thus, after acknowledging the expertise of the patient, the role of the clinician is to contribute information on each method contextualized by data specific to OUD. While participants feel clinicians who provide contraception counseling are generally familiar with the mechanism of action and common side‐effects of each method, they often feel that they are unable to ground their discussion in the unique needs and preferences of persons with OUD.

Our data highlight that regardless of specialty, the development of a trusting contraception counseling relationship is predicated on the clinician providing contraceptive information in the context of this chronic disease, as well as demonstrating the knowledge of pertinent reproductive health topics specific to OUD. To our knowledge, there is only one contraception information resource developed by professional societies specific to individuals with OUD.^26^ A focus on developing both clinician‐facing and patient‐facing evidence‐based contraception resources would allow for clinician engagement in a more informed dialogue with individuals with OUD and allow them to empower patients to gather evidence‐based contraception information in the context of their chronic disease.

One sequela of this lack of contraception information specific to individuals with OUD is that information sources outside the health care system are highly valued by this population. Individuals with chronic conditions are commonly excluded from contraception clinical trials, yet they are often actively engaged in disease‐specific internet threads and forums.^27^ Social media is used to provide social, emotional, and experiential support, with one study noting Facebook and blogs are more likely to improve, rather than harm care for people with chronic conditions.^28^ Consistent with prior studies of individuals with chronic conditions, no participants promoted the involvement of health care professionals in their social media forums.^29^ To our knowledge, there are no social media forums that discuss contraceptive evidence and experiences among individuals with OUD moderated by trained peer moderators. Training individuals already involved in these forums to be peer moderators would provide a patient‐led and accessible contraception intervention for individuals with OUD.

Our study also highlights the recovery home environment as a valued source of contraception information. Unlike other chronic conditions, individuals with OUD can exchange experiences and information on medical topics such as fertility, infectious disease, and medications by virtue of living together during the immediate postacute phase of substance use. Efforts to promote the dissemination of accurate information in the recovery home environment present a unique opportunity for intervention. Colocation of reproductive health services with OUD treatment has been successfully implemented in many outpatient treatment settings to meet the contraceptive information needs for individuals with OUD.^30^ Additionally, a recent study of a peer‐led, trauma‐informed contraception navigation intervention based in OUD treatment programs concluded this was feasible to implement, acceptable to participants, and showed preliminary evidence of efficacy in increasing family planning visits within diverse and resource‐limited treatment settings.^31^, ^32^ Our study further supports the acceptability and desirability of training peer contraception counselors in the recovery home.

Study strengths include being the first qualitative study to describe contraceptive information sources valued by persons with OUD, which is paramount to providing patient‐centered preventative reproductive health care. Additionally, qualitative interviews enabled us to obtain rich and nuanced perspectives from patients with OUD on their contraceptive experiences and preferred information sources. Our study does have limitations. Interviews were conducted in Massachusetts, where all Food and Drug Administration‐approved contraceptive methods are covered by Medicaid. Thus, our findings may not be generalizable to pregnancy‐capable individuals with OUD who have more limited contraceptive access. Additionally, our convenience sample may not reflect the perspectives of those who do not speak English or are not engaged in care. Our predominantly White non‐Hispanic patient population is reflective of ethnic disparities in the utilization of medication for opioid use disorder among pregnant individuals.^33^ We are thus unable to comment on the intersectional experiences of OUD and contraception care experienced by patients of different races and ethnicities. We also focused our sample on patients who were or had recently been pregnant but did not ask about pregnancy intention. It would be valuable to understand more about how contraceptive information received had or had not supported patients' fertility goals. Last, we collected these data prior to the onset of the COVID‐19 pandemic. Thus, our study does not address the health system changes, including decreased in‐person contact with the health care system, increased use of telemedicine with insurance reimbursement, and social distancing in recovery homes.^30^

In conclusion, an understanding of the contraception information sources valued by individuals with OUD may have significant implications for building therapeutic relationships and for the dissemination of accessible, evidence‐based, and trusted contraception information among individuals with OUD.

## FUNDING INFORMATION

This work was supported by a 2018 Society of Family Planning Emerging Scholars in Family Planning Grant [SFPRF12‐ES19].

## References

[1] Phillips‐Bell GS , Sappenfield W , Robbins CL , Hernandez L . Chronic diseases and use of contraception among women at risk of unintended pregnancy. J Women's Health. 2016;25(12):1262‐1269. doi:10.1089/jwh.2015.5576 PMC5154780 27295335

[2] Chor J , Rankin K , Harwood B , Handler A . Unintended pregnancy and postpartum contraceptive use in women with and without chronic medical disease who experienced a live birth. Contraception. 2011;84(1):57‐63. doi:10.1016/J.CONTRACEPTION.2010.11.018 21664511

[3] Finer LB , Zolna MR . Declines in unintended pregnancy in the United States, 2008–2011. N Engl J Med. 2016;374(9):843‐852. doi:10.1056/nejmsa1506575 26962904 PMC4861155

[4] Heil SH , Jones HE , Arria A , et al. Unintended pregnancy in opioid‐abusing women. J Subst Abus Treat. 2011;40(2):199‐202. doi:10.1016/j.jsat.2010.08.011 PMC3052960 21036512

[5] Terplan M , Kennedy‐Hendricks A , Chisolm MS . Article commentary: prenatal substance use: exploring assumptions of maternal unfitness. Subst Abus Res Treat. 2015;9s2(S2):SART.S23328. doi:10.4137/SART.S23328 PMC4578572 26448685

[6] Haddad LB , Monsour M , Tepper NK , Whiteman MK , Kourtis AP , Jamieson DJ . Trends in contraceptive use according to HIV status among privately insured women in the United States. Am J Obstet Gynecol. 2017;217(6):676.e1‐676.e11. doi:10.1016/J.AJOG.2017.08.006 28866122 PMC6674982

[7] Terplan M . Women and the opioid crisis: historical context and public health solutions. Fertil Steril. 2017;108(2):195‐199. doi:10.1016/j.fertnstert.2017.06.007 28697909

[8] Van Boekel LC , Brouwers EPM , Van Weeghel J , Garretsen HFL . Stigma among health professionals towards patients with substance use disorders and its consequences for healthcare delivery: systematic review. Drug Alcohol Depend. 2013;131(1–2):23‐35. doi:10.1016/J.DRUGALCDEP.2013.02.018 23490450

[9] Sobel L , Lee YW , White KOC , Woodhams E , Patton E . Contraceptive decision making among pregnancy‐capable individuals with opioid use disorder at a tertiary care center in Massachusetts. Contraception. 2021;104(4):355‐360. doi:10.1016/J.CONTRACEPTION.2021.06.002 34118268

[10] Bornstein M , Gipson JD , Bleck R , Sridhar A , Berger A . Perceptions of pregnancy and contraceptive use: an in‐depth study of women in Los Angeles methadone clinics. Womens Health Issues. 2019;29(2):176‐181. doi:10.1016/j.whi.2018.10.004 30446331 PMC6424631

[11] Daniell HW . Narcotic‐induced hypogonadism during therapy for heroin addiction. J Addict Dis. 2008;21(4):47‐53. doi:10.1300/J069V21N04_05 12296501

[12] Stancil SL , Miller MK , Duello A , et al. Long‐acting reversible contraceptives (LARCs) as harm reduction: a qualitative study exploring views of women with histories of opioid misuse. Harm Reduct J. 2020;18:83. doi:10.1186/s12954-021-00532-1 PMC8335991 34348734

[13] Terplan M , Hand DJ , Hutchinson M , Salisbury‐Afshar E , Heil SH . Contraceptive use and method choice among women with opioid and other substance use disorders: a systematic review. Prev Med (Baltim). 2015;80:23‐31. doi:10.1016/j.ypmed.2015.04.008 PMC4842019 25900803

[14] Mahony H , Spinner C , Vamos CA , Daley EM . Social network influences on young Women's choice to use long‐acting reversible contraception: a systematic review. J Midwifery Womens Health. 2021;66(6):758‐771. doi:10.1111/JMWH.13280 34491002

[15] Ali MM , Amialchuk A , Dwyer DS . Social network effects in contraceptive behavior among adolescents. J Dev Behav Pediatr. 2011;32(8):563‐571. doi:10.1097/DBP.0B013E318231CF03 21918469

[16] Choi KH , Gregorich SE . Social network influences on male and female condom use among women attending family planning clinics in the U.S. Sex Transm Dis. 2009;36(12):757‐762. doi:10.1097/OLQ.0B013E3181AFEFC1 19704396 PMC2787869

[17] Jawad A , Jawad I , Alwan NA . Interventions using social networking sites to promote contraception in women of reproductive age. Cochrane Database Syst Rev. 2019;2019(3):CD012521. doi:10.1002/14651858.CD012521.PUB2 PMC6395225 30818414

[18] Merz AA , Gutiérrez‐Sacristán A , Bartz D , et al. Population attitudes toward contraceptive methods over time on a social media platform. Am J Obstet Gynecol. 2021;224:597.e1‐597.e14. doi:10.1016/j.ajog.2020.11.042 33309562

[19] O'Connor AM , Stacey D , Jacobsen MJ . Ottawa decision support tutorial (ODST): improving Practitioners' decision support skills Ottawa Hospital Research Institute: patient decision aids. 2011:1–27. Accessed January 22, 2019. https://decisionaid.ohri.ca/ODST

[20] Harris PA , Taylor R , Thielke R , Payne J , Gonzalez N , Conde JG . Research electronic data capture (REDCap)‐‐a metadata‐driven methodology and workflow process for providing translational research informatics support. J Biomed Inform. 2009;42(2):377‐381. doi:10.1016/j.jbi.2008.08.010 18929686 PMC2700030

[21] Recovery Home Definition | Law Insider. Accessed April 26, 2022. https://www.lawinsider.com/dictionary/recovery-home

[22] Charron E , Mayo RM , Heavner‐Sullivan SF , et al. “It's a very nuanced discussion with every woman”: health care providers' communication practices during contraceptive counseling for patients with substance use disorders. Contraception. 2020;102(5):349‐355. doi:10.1016/j.contraception.2020.09.002 32941890

[23] Terplan M , Longinaker N , Appel L . Women‐centered drug treatment services and need in the United States, 2002–2009. Am J Public Health. 2015;105(11):e50‐e54. doi:10.2105/AJPH.2015.302821 26378825 PMC4605167

[24] Envision SRH PATH Questions . Accessed August 23, 2022. https://www.envisionsrh.com/path-framework

[25] White KO , Lerner NML , Raskin EJE . PHI CARE: A New Framework for Shared Decision‐Making in Contraception. Accessed September 12, 2022. https://picck.org/resource/phi-care-framework-and-video/

[26] Woodhams E , Samura T , White K , Patton E , Terplan M . Society of family planning clinical recommendations: contraception and abortion care for persons who use substances. Contraception. 2022;112:2‐10. doi:10.1016/j.contraception.2022.05.010 35644230

[27] Rezaallah B , Lewis DJ , Pierce C , Zeilhofer HF , Berg BI . Social media surveillance of multiple sclerosis medications used during pregnancy and breastfeeding: content analysis. J Med Internet Res. 2019;21(8):e13003. doi:10.2196/13003 31392963 PMC6702799

[28] Patel R , Chang T , Greysen SR , Chopra V . Social media use in chronic disease: a systematic review and novel taxonomy. Am J Med. 2015;128(12):1335‐1350. doi:10.1016/J.AMJMED.2015.06.015/ATTACHMENT/B4FEC07F-D235-4550-90C4-FEFD7BAB5299/MMC1.DOCX 26159633

[29] De Nardi L , Trombetta A , Ghirardo S , Genovese MRL , Barbi E , Taucar V . Adolescents with chronic disease and social media: a cross‐sectional study. Arch Dis Child. 2020;105(8):744‐748. doi:10.1136/ARCHDISCHILD-2019-317996 31941715

[30] Patton EW , Saia K , Stein MD . Integrated substance use and prenatal care delivery in the era of COVID‐19. J Subst Abus Treat. 2021;124:108273. doi:10.1016/j.jsat.2020.108273 PMC7979279 33771277

[31] Rinehart DJ , Stowell M , Collings A , et al. Increasing access to family planning services among women receiving medications for opioid use disorder: a pilot randomized trial examining a peer‐led navigation intervention HHS public access. J Subst Abus Treat. 2021;126:108318. doi:10.1016/j.jsat.2021.108318 PMC8197777 34116817

[32] Stowell MA , Thomas‐Gale T , Jones HE , Binswanger I , Rinehart DJ . Perspectives among women receiving medications for opioid use disorder: implications for development of a peer navigation intervention to improve access to family planning services. Subst Abus. 2022;43(1):722‐732. doi:10.1080/08897077.2021.2007514 35100081 PMC9743830

[33] Schiff DM , Nielsen T , Hoeppner BB , et al. Assessment of racial and ethnic disparities in the use of medication to treat opioid use disorder among pregnant women in Massachusetts. JAMA Netw Open. 2020;3(5):e205734. doi:10.1001/JAMANETWORKOPEN.2020.5734 32453384 PMC7251447

